# Relevance of the Diversity among Members of the *Trypanosoma Cruzi* Trans-Sialidase Family Analyzed with Camelids Single-Domain Antibodies

**DOI:** 10.1371/journal.pone.0003524

**Published:** 2008-10-24

**Authors:** Laura Ratier, Mariela Urrutia, Gastón Paris, Laura Zarebski, Alberto C. Frasch, Fernando A. Goldbaum

**Affiliations:** 1 Instituto de Investigaciones Biotecnológicas-Instituto Tecnológico de Chascomús (IIB-INTECH), Universidad Nacional de General San Martín-CONICET, Buenos Aires, Argentina; 2 Fundación Instituto Leloir, Instituto de Investigaciones Bioquímicas Buenos Aires-CONICET, Buenos Aires, Argentina; Case Western Reserve University, United States of America

## Abstract

The sialic acid present in the protective surface mucin coat of *Trypanosoma cruzi* is added by a membrane anchored trans-sialidase (TcTS), a modified sialidase that is expressed from a large gene family. In this work, we analyzed single domain camelid antibodies produced against trans-sialidase. Llamas were immunized with a recombinant trans-sialidase and inhibitory single-domain antibody fragments were obtained by phage display selection, taking advantage of a screening strategy using an inhibition test instead of the classic binding assay. Four single domain antibodies displaying strong trans-sialidase inhibition activity against the recombinant enzyme were identified. They share the same complementarity-determining region 3 length (17 residues) and have very similar sequences. This result indicates that they likely derived from a unique clone. Probably there is only one structural solution for tight binding inhibitory antibodies against the TcTS used for immunization. To our surprise, this single domain antibody that inhibits the recombinant TcTS, failed to inhibit the enzymatic activity present in parasite extracts. Analysis of individual recombinant trans-sialidases showed that enzymes expressed from different genes were inhibited to different extents (from 8 to 98%) by the llama antibodies. Amino acid changes at key positions are likely to be responsible for the differences in inhibition found among the recombinant enzymes. These results suggest that the presence of a large and diverse trans-sialidase family might be required to prevent the inhibitory response against this essential enzyme and might thus constitute a novel strategy of *T. cruzi* to evade the host immune system.

## Introduction

Unicellular eukaryotic pathogens have developed a variety of mechanisms to survive in the multicellular organisms that they parasitize [1]. Some of these mechanisms involve surface/shed molecules required to invade cells from the host and/or to evade the host immune response. The human pathogen *Trypanosoma cruzi*, the agent of the American endemic Chagas disease, has two essential mechanisms to survive in the mammalian host: an intracellular stage and the presence of a diverse surface membrane coat (reviewed in [2]). This coat is made of mucins that are highly glycosylated proteins expressed from a large gene family (reviewed in [3]). The coordinate expression of a large repertoire of mucins containing variable regions in the mammal stages of the *T. cruzi* life cycle suggests a possible strategy to delay the host immune response [3]. The mucin sugar moiety contains sialic acid that is implicated in key aspects of parasite-host interactions such as cell adhesion and invasion [4], and resistance to non-specific complement attack [5]. Since trypanosomes are unable to synthesize sialic acid, sialylation of mucins is possible due to the activity of *T. cruzi* trans-sialidase (TcTS), a modified sialidase that instead of hydrolyze sialic acid, transfer the sugar from host glycoconjugates to α-galactoses present in mucins of the parasite surface (reviewed in [6]). The three-dimensional structure and the catalytic mechanism of the enzyme were recently determined [7]–[10]. TcTS has a globular core with two domains connected by a long α-helix. The N-terminal domain has a six bladed β-propeller fold and contains the catalytic site. The C-terminal domain shows a lectin-like topology and has not any activity reported until now. In addition to the globular core of the protein, there is a variable number of C-terminal highly antigenic 12 amino acid repeated motif known as SAPA (shed acute phase antigen) [6], [11]. This motif allows the enzyme to remain in blood [12], [13]. Strong anti-SAPA humoral immune response is observed during the acute phase of Chagas' disease [14], [15]. TcTS is encoded in a large gene family of about 140 members, the protein products differing by about 5% in their primary sequence. Half of the gene family members code for inactive proteins due to a mutation in the active site nucleophile Tyr342 by a His [16], [17]. In addition, there are about 1000 genes that were named “trans-sialidase-like” because they have about 30–80% of identity to trans-sialidase genes but lack enzymatic activity [6]. TcTS is a relevant factor in the infection and pathogenesis of *T. cruzi*. Recently, it has been demonstrated that TcTS is responsible of inducing transient thymic aplasia via apoptosis. This effect could allow the avoidance of the host immune system by the parasite [18]. Given the essential roles of TcTS in infection and pathogenesis, this enzyme is a good target for the development of alternative chemotherapy agents against the parasite. Nevertheless, small compounds with high inhibitory activity for trans-sialidase are not currently available. A sialidase inhibitor, 2,3-didehydro-2-deoxy-N-acetylneuraminic acid (DANA), an analog to the oxocarbenium transition state of the reaction, is 100-fold less effective toward TcTS (Ki = 10 mM) than to bacterial and viral sialidases [19]. Characterization of alternative acceptor molecules, lactose derivatives, allowed the finding of lactitol. This monosaccharide is a better sialic acid acceptor than conventional substrates like lactose. Lactitol partially prevents parasite sialylation and invasion into host cells [20] and the apoptotic effect of TcTS on cells of the immune system [21].

Antibodies able to neutralize TcTS activity are normally found in patients with chronic Chagas' disease and animals infected with *T. cruzi* [15], [22], [23]. However, the onset of this antibody response is delayed until the end of the acute phase and coincides with a decline in parasitemia levels [15]. Anti-trans-sialidase activity has been detected in the sera of infected patients using a very specific and sensitive assay, named trans-sialidase inhibition assay (TIA) [24]. A monoclonal antibody that neutralizes TcTS activity was recently obtained. Passive transfer of this antibody to infected animals prevents TcTS induced thrombocytopenia [25].

Despite the large structural and functional diversity of the mammalian antibody repertoire, conventional antibodies (i.e. heterotetramers of two light chains and two heavy chains) acting as competitive enzyme inhibitors are scarcely found in bibliography. They recognize enzymes (and globular proteins in general) by flat complementary surfaces composed by loops of both the heavy and light variable domains. As the active site of TcTS is located in a deep cleft of the protein, it is difficult to obtain a convex binding surface to reach it by experimental immunizations that elicit conventional antibodies [25], [26]. Camelidae, besides the conventional antibodies, also express heavy-chain antibodies, homodimers that consist of only heavy chains [27]. Their variable region, named VHH, is the smallest natural antigen-binding fragment (∼16,000 Da), and being just one polypeptide chain it is especially suitable for engineering. In particular, longer complementarity-determining region 3 (CDR3) loops protruding from the binding site and the deviation of CDR conformations from the equivalent human or mouse loop structures, suggest that camelid single domain antibodies might have different strategies of binding [26], [28]. In contrast to the antigen binding fragments of conventional antibodies, VHHs are often potent inhibitors of enzymes [26], [28]. Some long CDR3 loops protruding to the active site of the enzyme are responsible for that inhibition.

In this work we show that inhibitory antibodies against trans-sialidase can be obtained by phage display selection of single-domain antibody fragments from immunized llamas. These antibodies inhibited the recombinant TcTS that was used for immunization. However, they were unable to inhibit partially purified TcTS from *T. cruzi* parasites, which are naturally expressed from different genes. Our results suggest that subtle mutations in members of the TcTS family prevent the complete neutralization of the parasite enzymatic activity.

## Results

### Immunized llamas show polyclonal inhibitory response against TcTS activity in sera

**Figure 1:**
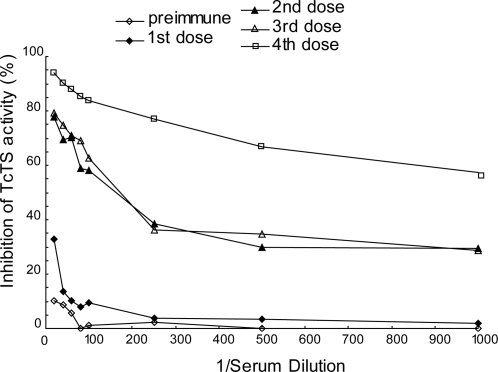
Inhibition of TcTS activity by llama 7006 serum. Serial dilutions of pre-immune and post-recombinant TcTSΔ1443-SAPA immunization sera, were analyzed by TIA as indicated under Materials and Methods.

Two llamas were immunized using different recombinant TcTS constructions. Llama 7006, was immunized with pTcTSΔ1443 (lacking the 1443 epitope and retaining the SAPA repeats). This recombinant protein was used since deletion of the internal epitope between amino acids 433 and 447, called epitope 1443, increases the production of neutralizing antibodies in mouse models of infection [29], [30]. The second camelid, named llama 9210, was immunized with protein from the clone pTrcTS611/2 (entire globular core of TcTS without SAPA repeats) [31]. Llama 9210 showed a late TcTS inhibitory response and at lower level than llama 7006 (data not shown). Due to the high polyclonal inhibitory response detected in serum from llama 7006 after the fourth immunization, we engaged in the construction of a VHH library from the RNA of lymphocytes isolated from this animal fifteen days after the last immunization (Figure 1). The absence of 1443 epitope and/or the presence of SAPA repeats that increase the half-life in blood could be responsible for the difference in the inhibitory response between both llamas.

### Screening for inhibitory clones

**Figure 2:**
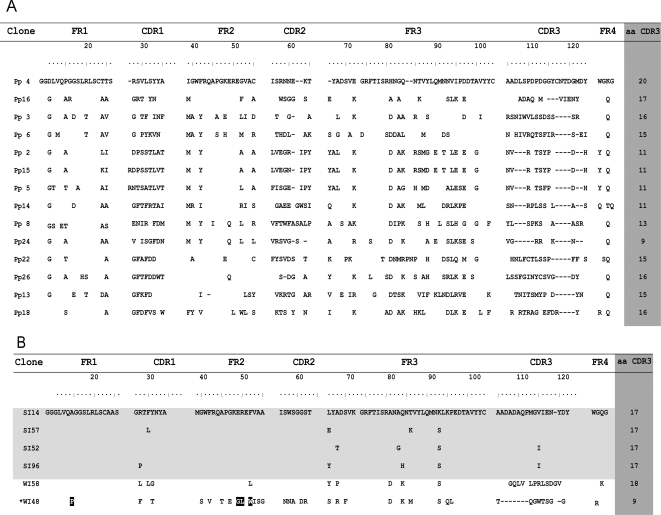
(A) *Alignment of the sequences of 14 randomly chosen clones from the library before panning* (pre-panning clones are labeled pp) and (B) *Sequence analysis of the six post-panning selected VHHs*.The 4 strong inhibitors (SI) clones are shown in clear gray background. Lengths in CDR3 are shown in dark gray background. Clone WI48 is a conventional IgG (*) and its characteristic residues are marked in black. Spaces denote identical residues and dashes denote deletions. Numbering and CDR designations are according to IMGT numbering system [46].

**Figure 3:**
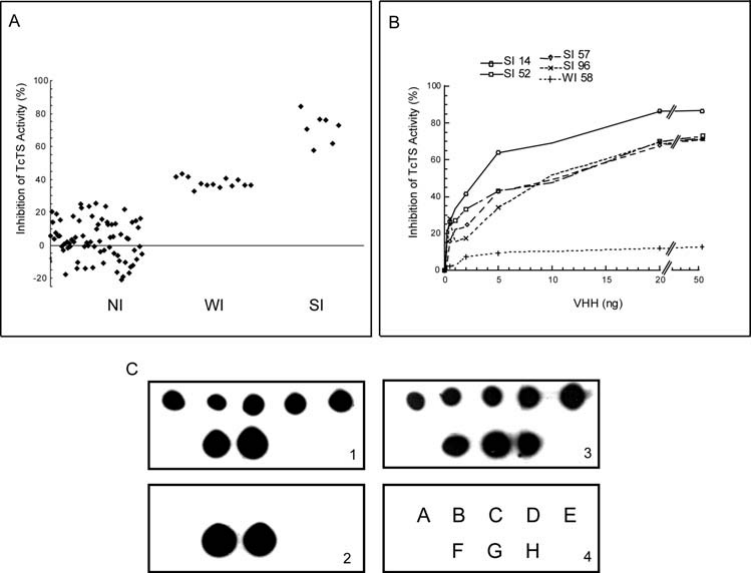
(A) *Screening of VHH library*. 94 individual phages-VHH clones were tested by TIA using TcTS611/2. Three clear groups are observed, NI: non-inhibitors, WI: weak inhibitors, SI: strong inhibitors. Each point represents phages prepared from a single colony. TIA values lower than 25% were considered as negative. (B) *Purified VHHs inhibit recombinant TcTS activity*. A fixed mass of 0.5 ng of purified TcTS611/2 was preincubated with increasing concentrations of each VHH and trans-sialidase activity was analyzed by TIA. The values represent the average of at least three independent determinations. (C) *The selected VHHs recognize conformational epitopes*. Recombinant proteins, all carrying a His-tag, were spotted onto nitrocellulose membrane as indicated in the panel 4: A) SI14, B) WI58, C) SI52, D) SI96, E) SI57, F) TcTS611/2, G) Denatured TcTS611/2 in 0.1% SDS, H) Non-related (non-anti-TcTS) VHH. Panel 1 and panel 2 were incubated with native TcTS and denatured TcTS in 0.1% SDS, respectively. Both panels were treated with rabbit anti-TcTS serum. Panel 3 was processed with a mouse anti-Histidine antibody as a control for protein immobilization. Filters were revealed with the corresponding HRP-conjugated-anti-serum for chemiluminescence generation.

The quality of the library, composed by 2×10^6^ single clones, was checked by sequencing fourteen randomly chosen clones, which showed high variability in nucleotide sequence (Figure 2A). Ninety-four phage-VHH clones obtained from the first round of panning were analyzed by TIA (trans-sialidase inhibition assay, see Materials and Methods) using TcTS611/2. This preliminary TcTS inhibition screening allowed us to identify three clearly defined groups of VHHs: non-inhibitors (NI), weak inhibitors (WI) and strong inhibitors (SI) represented by 74, 13 and 7 clones, respectively (Figure 3A). Since the total percentage of inhibitors (21%) is smaller than the percentage of binders (71%, Table 1), the use of a screening inhibition assay resulted in a powerful strategy to select phage-VHH for inhibitory clones. This result was reproducible when testing soluble VHHs (results not shown). After sequencing, the seven strong TcTS inhibitor clones showed to correspond to four unique clones (clones SI14, SI52, SI57 and SI96, Figure 2B). Selected weak inhibitor clones WI58 and WI48 (a conventional IgG VH) were included for further comparison.

**Table 1:** Panning of the library and its evaluation by phage-ELISA.

	1^st^ round	2^nd^ round	3^rd^ round
Input	7.3×10^12^	1.6×10^13^	8.2×10^12^
SBO^a^	3.8×10^8^	3.7×10^10^	1.6×10^10^
NSBO^b^	3.4×10^7^	2.4×10^10^	1.0×10^9^
TcTS binders/total clones	10/14 (71%)	16/32 (50%)	3/30 (10%)

a SBO: specific binding output.

b NSBO: Non-specific binding output

Numbers are expressed as colony forming units.

As shown in figure 2, none of the SI selected clones appear among the sequenced prepanning clones (except for prepanning clone 16), indicating that their selection was not due to overrepresentation in the library. Besides, prepanning clones showed a higher diversity in sequence and in the CDRs length than inhibitory selected clones. Particularly, CDR3 of prepanning clones range from 9 to 20 amino acids, meanwhile SI clones share the same CDR3 length (17 residues). A common characteristic of VHHs is the presence of long CDR3 loops, which confer increase diversity to the binding site of these single domain antibodies. Clones displaying strong inhibition capacity had very similar sequences, showing that they derived, likely, from the same original clone that had undergone different somatic mutations that affect a pair of CDR residues and framework region 3. In contrast, clones WI48 and WI58, which had low inhibition capacity, had different CDR3 sequence and length, indicating that derived from different clones.

### Single domain llama antibodies that inhibit recombinant TcTS activity recognize a conformational epitope with affinities in the nanomolar range

Figure 3B shows the inhibitory activity displayed by soluble purified VHHs against recombinant TcTS611/2. Clone SI14 showed the strongest inhibition activity while clone WI58 showed lower TcTS inhibition activity. We measured the affinities of VHH-TcTS complexes using a biosensor (Table 2). The four selected SI clones showed similar affinities for TcTS, with K_D_ values in the high nanomolar range (22 to 86 nM). Thus, there is a correlation between affinity and inhibition capacity among VHHs since clone SI14, which showed the higher affinity to TcTS on biosensor analysis has the higher inhibition capacity. To analyze whether inhibitory VHH recognize conformational or linear epitopes, purified VHHs spotted onto a nitrocellulose membrane were reacted with native and denatured recombinant TcTS611/2. All VHHs bound native TcTS but did not recognize denatured TcTS (Figure 3C) indicating that the selected inhibitory VHHs recognize discontinuous TcTS epitopes.

**Table 2:** Affinities of TcTS-VHH complexes (IAsys biosensor analysis).

TcTS	VHH clone	*Kon* (10^5^ M^−1^s^−1^)	*Koff* (10^−3^ s^−1^)	*KD* (10^−9^ M)^a^
611/2	SI14	1.19±0.10	2.65±0.04	22.27±0.24
	SI52	1.49±0.05	10.30 ±0.24	69.03±0.43
	SI57	1.65±0.13	14.27 ±0.20	86.34±1.92
	SI96	3.52±0.28	17.05±0.21	48.44±0.44
Trp312Ala	SI14	0.62±0.04	14.50±0.30	230.10±17.22
Try119Ser	SI14	0.74±0.03	14.70±0.30	197.16±12.11

a The K_D_ value was determined as the K_off_/K_on_ ratio.

### Strong inhibitor VHHs recognize an epitope overlapped to the active site

Tyr119 and Trp312 are key residues for the activity of the enzyme since proteins mutant TcTS Tyr119Ser and Trp312Ala loose 90% and 100% of transfer activity, respectively [9], [10], [32]. We measured the binding affinity of VHHs to mutant TcTSTrp312Ala and TcTSTyr119Ser. VHHSI14 binds to both mutants with approximately ten times lower affinity compared to recombinant TcTS611/2 (Table 2), indicating that VHHSI14 recognizes residues near or in the active site of TcTS.

**Figure 4:**
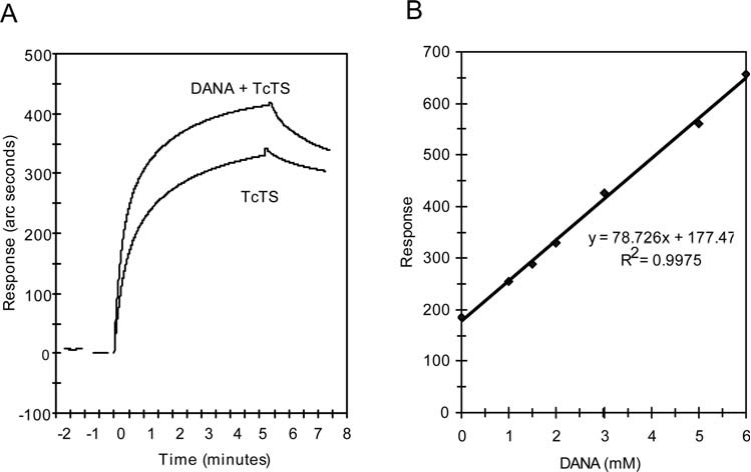
DANA increased the VHH binding to recombinant TcTS. Soluble TcTS611/2 was added in the presence or the absence of DANA to a cuvette derivatized with VHH. (A) Binding kinetics of VHHSI96 to TcTS in the presence or absence of 10 mM DANA. (B) Response at the equilibrium of VHHSI14-TcTS in different concentrations of DANA. The addition of DANA did not affect the baseline.

The decrease in the binding affinity of VHHSI14 to mutant TcTS prompted us to test the effect of the conformational change of Tyr119. Upon binding of DANA to TcTS active site, residue Tyr119 moves away from catalytic center and it is positioned in front of the indole ring of Trp312 [8], [33]. To study if llama inhibitory single domain antibodies sense the TcTS conformational change produced by DANA, we analyzed the binding kinetics and the response at the equilibrium in the presence or the absence of this ligand. As seen in Figure 4A, the signal obtained upon TcTS611/2 binding increased in the presence of DANA. This increment in the signal is similar to the effect of increasing ten times the concentration of TcTS. As shown Figure 4B, there is a linear correlation between the increase in VHHSI14-TcTS binding response and the DANA concentration. This effect was similarly observed in all the four SI VHHs selected clones (data not shown). This result implies a higher affinity of the SI clones for the conformation adopted by the enzyme upon binding of DANA, which would then enhance the SI VHHs competitive inhibition activity. Interestingly that sialic acid is present at high concentrations in blood [34], thus it is possible that the immune system recognize TcTS in this particular acceptor-bound conformation. In summary, we obtained different results suggesting that this family of single domain llama antibodies recognize with high affinity an epitope close or overlapped to the active site of the enzyme.

**Figure 5:**
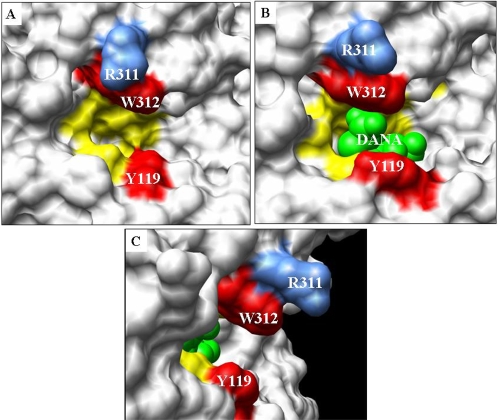
The epitope recognized by inhibitory llama antibodies maps to the TcTS catalytic site. View of the TcTS active site shown as surface representation using the program Chimera. (A) Free TcTS, (B) DANA-TcTS (shows the conformational change upon binding of DANA to TcTS) and (C) shows a 90° rotation of TcTS-DANA structure highlighting the arginine 311 residue protruding from the active site. PDBs used are 1MS4 and 1MS8. Residues involved in the catalytic site are colored as follows: mutated residues that were analyzed in this work (Trp312 and Tyr119) in red, other catalytic amino acids (Arg35, Asp59, Asp96, Met98, Arg314, Arg245, Glu230 and Tyr342) in yellow, space-fill model of DANA in green and Arg311 in blue.

Epitope mapping analysis of the binding of the SI VHHs to recombinant TcTS is shown in Figure 5. Comparing free TcTS and DANA-TcTS structures (Figure 5A and 5B), it can be seen that Tyr119 suffers a conformational change upon binding to DANA [8], [33].

### Single domain antibodies fail to inhibit the natural trans-sialidase

**Figure 6:**
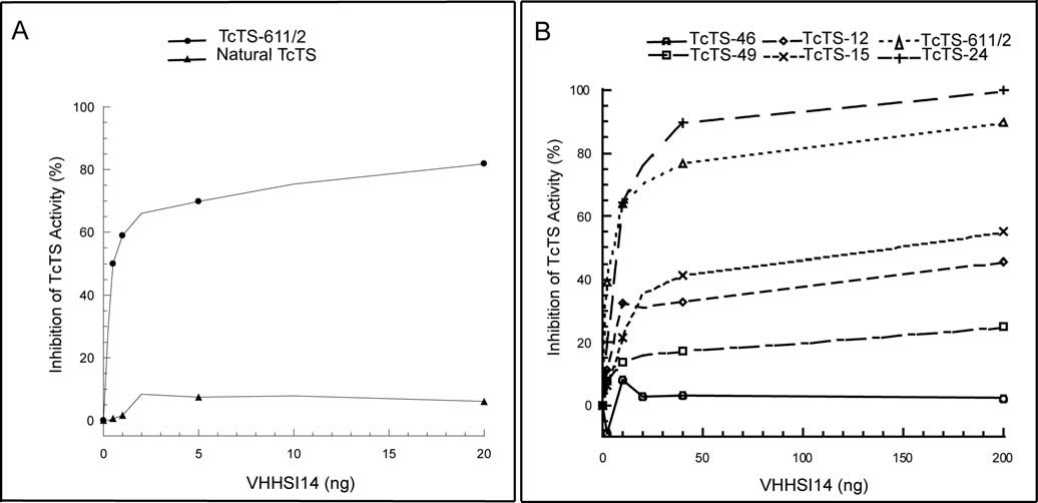
(A) *Natural TcTS was not inhibited by VHHSI14.* Comparison of VHH114 inhibition activity of the TcTS present in supernatants from Cl-Brener *T. cruzi-*infected cell cultures versus a recombinant TcTS611/2 enzyme. The decameric form of VHHSI14 was used. The values represent the average of two independent determinations. (B) *Independent recombinant TcTSs are inhibited to different extents by VHHSI14*. A fixed mass of 0.5 ng of different purified TcTSs from *T. cruzi* were preincubated with increasing concentrations of VHHSI14 decameric form and the remaining trans-sialidase activity was analyzed by TIA. The values represent the average of at least three independent determinations.

VHHSI14 was tested against supernatants of Cl-Brener *T. cruzi*-infected cell cultures containing TcTS released from the trypomastigote stage of the parasite. Surprisingly, VHHSI14 failed to inhibit the enzyme present in parasite supernatants while a strong inhibition activity toward the recombinant TcTS611/2 used as a control was observed (figure 6A). Supernatants from three different strains were assayed in the presence of an excess of VHHSI14 antibody, showing similar results (Table 3). The lack of inhibition by VHHSI14 antibody was not due to the presence of any compound in the medium since recombinant TcTS added in the reaction was neutralized (Table 3). Similar negative results were observed with live Cl-Brener trypomastigotes containing TcTS linked to the membrane surface of the parasites (data not shown). To analyze if the ausence of inhibition was due to the univalent nature of VHHs, we increased the avidity of this antibody fragment. To this end, we constructed a fusion protein displaying ten VHH domains per assembly, taking advantage of the decameric structure of *Brucella spp*. lumazine synthase [35]. This assembly allowed to increase 10 times the avidity of VHHSI14 for recombinant TcTS but did not show differences in its inhibitory capacity as compared with that of the monomeric VHHSI14 (data not shown). This construction was assayed against Cl-Brener trypomastigotes and it was still unable to inhibit the activity of TcTS present in the surface of parasites (Table 3).

**Table 3:** Effect of VHHSI14 on natural TcTS from T. cruzi trypomastigotes.

	TcTS activity*^a^*		
	Total activity	Preincubated with VHHSI14 (40 ng)	Preincubated with lactitol 1 mM^b^
Recombinant TcTS611/2^c^	3279±314	621±212 **(81.1)** d	422±218 **(87.1)**
Natural TcTS^e^			
Cl-Brener strain	1154±135	1017±71 **(11.9)**	188±33 **(83.7)**
RA strain	1653±184	1576±162 **(4.6)**	**ND** f
Villegas strain	1960±90	1562±145 **(22.8)**	514±70 **(73.7)**
Natural TcTS plus recombinant TcTS			
Supernatant (Cl-Brener)	2318±288	2082±272 **(10.2)**	**ND**
Recombinant TcTS611/2	3286±401	651±201 **(80.1)**	**ND**
Supernatant (Cl-Brener) plus recombinant TcTS611/2	5134±109	2461±466 **(52.1)**	**ND**
Cl-Brener trypomastigotes g	918±168	834±102 **(4.8)**	**ND**
Inmunoprecipitated TcTS (Cl-Brener) g			
Treated with PNGasa	2632±396	2411±450 **(9.2)**	298±198 **(88.7)**
Untreated with PNGasa	2157±237	2079±243 **(3.6)**	321±184 **(85.1)**

a Results are expressed in CPM (Counts Per Minute) obtained after 1 hour of reaction (by TIA). The values are the mean and standard deviation of at least three independent experiments.

b Lactitol was assayed as positive inhibition control on Natural TcTS.

c Recombinant TcTS was assayed as positive control of inhibition in every single test.

d Parenthesis indicate percentage of inhibition of TcTS activity that remnant CPM represents .

e Supernatant from cell culture derived trypomastigotes were used.

f ND: not determinated.

g Decameric form of VHHS14 was used.

### The lack of inhibition of the natural trans-sialidase is due to differences among members of the enzyme family

A possible explanation for this contradictory result could be the existence of post-translational modifications present in the natural enzyme but absent in the recombinant *E. coli* TcTS enzymes. It is known that natural TcTS is glycosylated. One of the two possible N-glycosylation sites, located in residue 114, is close to the active site (predicted by NetNgly 1.0 server, see figure S2) and it corresponds with a N-glycosylation observed in residue 115 of *Trypanosoma rangeli* sialidase [8], [9]. No VHHSI14 inhibitory activity was seen when natural TcTS was immunoprecipitated from extracts of Cl-Brener trypomastigotes (the infective form of the parasite in the mammalian host) using an anti-SAPA antibody and treated with the high processive enzyme PNGase F. Controls of the deglycosylation process are shown in figure S1. Thus, it is unlikely that the presence of sugars in the natural TcTS were the reason of the lack of inhibition by VHHSI14 (Table 3).

*T. cruzi* is known to have a large number of TcTS genes, 70 in a strain of the parasite, expressing enzymatically active proteins [16]. It is also known that these genes somewhat differ in their primary sequence. These differences might cause the variations in the inhibitory effects of antibodies on the TcTS family. To test this hypothesis, we cloned and expressed five active recombinant TcTS clones by PCR (see sequences in Figure S2, supplemental data) using specific primers to amplify the entire globular core of the enzyme (see Materials and Methods). Figure 6B resume results of VHHSI14 decameric form tested toward these recombinant TcTSs clones using TIA assay. The five clones plus the TcTS611/2, used as a control, were inhibited to different extents by the VHHSI14 when they were tested under the same experimental conditions. TcTS-46 and TcTS-49 were poorly inhibited when increasing amounts of VHHSI14 were added. Both clones have several changes in their primary sequence as deduced from DNA sequencing (90.3 and 90.6% of identity respect TcTS611/2, see Table S1 in supplemental data). TcTS-24 was highly inhibited, even more than TcTS611/2, the clone whose product was used for the immunization, while TcTS-12 and TcTS-15 present intermediate inhibitory levels. Similar results were obtained when an excess of the other strong inhibitors VHHs were tested with TcTSs (Table 4). These results suggest that the VHHs antibody fragments might recognize minor antigenic differences between natural TcTSs resulting in different inhibition levels.

**Table 4:** Effect of 200 ng of each strong inhibitor VHH on different TcTS clones from Cl-Brener strain.

	TcTS activity*^a^*					
	TcTS-24	TcTS-611/2	TcTS-15	TcTS-12	TcTS-49	TcTS-46
Total TcTS Activity	208±16	1080±53	1085±110	1195±107	506±71	260±59
VHHSI14-decameric form	5±4 **(97.7)** b	218±28 **(79.8)**	522±83 **(51.9)**	606±60 **(49.3)**	368±85 **(27.3)**	238±70 **(8.4)**
VHHSI14	4±4 **(98.0)**	143±51 **(86.7)**	320±105 **(70.6)**	480±43 **(59.9)**	237±77 **(53.1)**	158±42 **(39.1)**
VHHSI52	4±4 **(98.0)**	202±37 **(81.3)**	340±59 **(68.7)**	512±85 **(57.2)**	258±135 **(49.0)**	159±47 **(38.9)**
VHHSI57	3±3 **(98.4)**	181±31 **(83.3)**	204±13 **(81.2)**	512±55 **(57.2)**	248±93 **(51.1)**	177±85 **(31.7)**
VHHSI96	12±8 **(94.2)**	211±63 **(80.5)**	354±71 **(67.4)**	485±44 **(59.5)**	383±90 **(24.4)**	170±82 **(34.8)**
Lactitol 1mM^c^	13±13 **(93.9)**	165±72 **(84.7)**	175±69 **(83.9)**	193±39 **(83.8)**	52±37 **(89.8)**	21±20 **(92.2)**

a Trans-sialidase activity was expressed in nmoles of syalil residue transferred to lactose×mg^−1^×min^−1^ (by TIA). The values are the mean and standard deviation of at least three independent experiments.

b Parenthesis indicate percentage of inhibition of TcTS activity that the remnant activity represents.

c Lactitol was used was assayed as positive control of inhibition of TcTS activity.

Next we analyzed the identity of the amino acid residues in the active site among all TcTS clones tested. All TcTSs that were inhibited by the camelid antibody have an arginine at position 311, which protrudes from the active site (as is shown in the wild-type TcTS-DANA complex structure, Figure 5C). In contrast, both TcTS that were almost not inhibited by VHHSI14 (TcTS-46 and TcTS-49) have a tryptophan residue in position 311 instead of arginine. We postulate that this bulky tryptophan, among other differences, might interfere with the binding to antibodies. Since we found that two mutations at single amino acid positions strongly affect VHH binding (Table 2), these results are in agreement with the possibility that differences in amino acid residues located close to, or in, the active site might result in the lack or decreased inhibition of some TcTS members by the llama antibodies.

## Discussion

In this work, we report the identification of single domain llama antibodies against recombinant TcTS that bind a site close or overlapping the active site of the enzyme. The antibodies had the expected inhibitory capacities and affinities and were obtained after one round of phage display selection. Three key factors allowed the success of the strategy used. First, the immunization of llamas allowed us to obtain the smallest antibody fragments with usually protruding long loops that facilitate the access to the active site cleft of enzymes. Second, the use of engineered TcTS, as immunogen, that increased the probabilities of raising antibodies that recognize the enzyme active site. Third, the use of a strategy that is based on a soluble inhibition screening test, instead of the classic binding assay to select a phage display library. Clones showing strong inhibitory activities represent approximately 7% of the selected clones, which is a logical result taking into account that TcTS is a large protein (70,000 Da). Thus, a sensitive phage-TIA assay allowed us for a simple, fast and very efficient selection of clones with inhibitory capacity. All seven selected clones represent four different single domain antibodies derived from the same clone, as can clearly be seen for their high sequence homology and an identical VDJ recombination sites. The consensus sequence contains a 17 residues long CDR3, with just one conservative difference at position 115 (Valine to Isoleucine). Thus, these results suggest that in our library there is only one structural solution for obtaining antibodies tightly binding to the recombinant TcTS active site used as immunogen.

VHHs described in the literature recognize globular proteins with affinities between 20–100 nM [36]. Particularly, camelid fragments with enzymatic inhibitory capacities [26], [36]–[38] have affinities in the 25±21 nM range, showed similar CDR1 and CDR2 length (8±1.5 and 7.7±0.7 residues, respectively) and differ in the number of disulfide bonds and in the CDR3 length from 11 to 24 residues, resulting in an average of 15.8±3.6 residues. Thus, our four selected clones fall within the range of the previously described inhibitory single domain antibodies since they showed affinities in the 22–86 nM range and have CDR lengths of 8, 7 and 17 residues for CDR1, CDR2, and CDR3, respectively. As the four selected VHHs derive from the same clone, thus it is fair to assume that all of them bind to the same epitope on TcTS. In coincidence, there is a clear correlation between affinity and inhibitory capacity among these four clones, being clone SI14 the one that shows the higher affinity and also the stronger inhibition capacity.

Site directed mutagenesis of TcTS Tyr119 and Trp312 residues, which are implicated in the enzymatic mechanism of trans-sialidase activity, decreases about ten times their binding to the antibodies (Table 2), while the addition of DANA increases their binding to TcTS (Figure 4). It can be inferred that these antibodies recognize this surface on the catalytic site. Whether the longer CDR3 loops of these antibodies penetrate inside the cavity or, alternatively, the binding of the VHHs does not allow the access of the acceptor lactose, remains to be demonstrated.

Despite we observed a strong inhibition of the recombinant TcTS used for immunization by llama single domain antibodies; they were unable to inhibit the complex mixture of TcTSs expressed in trypomastigote parasites. Purified VHHs inhibited recombinant TcTS611/2 by 60–80% and VHHS114 inhibited 8–98% the enzymatic activity of different recombinant trans-sialidases. On the other hand, the natural enzyme from Cl-Brener strain was inhibited only between 3% and 12%. There are several possible explanations for these apparently contradictory findings. One is that glycosylation of the natural enzyme, not present in the enzyme expressed in bacterial systems, might prevent the interaction of the enzyme with the VHH. This does not seem to be the case (see Fig. S1). However, we can not completely exclude the possibility that PNGase failed to fully deglycosylate all of the different natural TS enzymes. Even if this was the case, these results do not invalidate those showing that different recombinant enzymes expressed in bacterial systems are inhibited to different extents by VHHs, one of the main conclusions of this work. An alternative explanation is the presence of TcTS-like molecules lowering the effective concentration of VHHs or the presence of other inhibitors in the natural enzyme sample. A recombinant TcTS added to the natural enzyme sample was inhibited, precluding the previously mentioned possibility. Clearly, it is not possible at present to find a simple explanation for the above mention findings, which awaits results from a different approach, like structural studies, to solve it.

The results obtained in this work, showing that different recombinant TcTS protein products are inhibited to different extents by the four VHHs tested, indicate that VHHs could recognize minor antigenic differences present in the polymorphic population of natural trans-sialidases, as this enzymatic activity potentially derives from the simultaneous expression of about 70 different genes [39]. These observations suggest an interesting hypothesis to explain the possible usefulness for the presence of a TcTS family. If trans-sialidase activity results from the expression of a single gene, one antibody clone might be enough to completely neutralize the enzymatic activity. The simultaneous expression of a large number of trans-sialidases slightly differing in primary sequence but not in enzymatic activity might delay the complete inhibition of the parasite enzyme. In addition to genes encoding trans-sialidase enzymes, there is a larger number of trans-sialidase genes (about 1000), coding for proteins with homologies to trans-sialidase but lacking enzymatic activity. These non-enzymatic proteins might be further involved in the distraction of the immune antibody response against active members of the family [39]. In summary, a large part of the genome is devoted to encode trans-sialidase and trans-sialidase-like proteins likely to be involved in the protection against a neutralizing activity that might prevent parasite development in the mammalian host.

Presently, we do not know which TcTS amino acid positions are recognized by the llama antibody VHHSI14 to neutralize enzymatic activity. Future work, including the determination of TcTS structure in complex with antibodies will indicate if there are mutational hot-spots for the generation of TcTS diversity preventing interaction with antibodies but not affecting the enzymatic activity. However, structural and bioinformatic analyses allowed us to identify a key role of residue 311. Arg311 side chain protrudes in the active site while both poorly inhibited TcTS (TcTS-46 and TcTS-49) have a bulky tryptophan at this position. Thus, we postulate that Trp311 would be responsible for steric hindrance of the VHH binding to the epitope recognized in the enzyme.

We have previously postulated that the amplification and divergence of the members of the mucin family covering the trypanosomal surface are also involved in delay the host immune response [3]. In this case, coordinate expression of a large repertoire of mucins containing variable regions in the mammal stages of the *T. cruzi* life cycle might delay a lytic antibody response. In the case of TcTS, coordinate expression of several enzymatically active proteins having subtle differences at amino acid positions in or around the active site might delay the immune response inhibiting the complete enzymatic makeup of the parasite. Antibodies neutralizing TcTS are detectable in the host serum later during the infection with *T. cruzi* [15], [24], and through immunization with recombinant enzymes or DNA [21], [25], [40]–[43]. Thus, our model of epitope variation among TcTS members to prevent an inhibitory response might apply for the early stages of the infection, period during which the parasite requires the necessary time to reach the niches inside the host cells.

## Materials and Methods

### 

#### TcTS expression and purification

*E. coli* strain BL21 (DE3) pLysS (Novagen) was transformed with plasmids (pTrcHisA, Invitrogen) encoding different TcTS clones. A dilution of the over night culture was grown until A_600nm_ 1.0–1.2 in constant agitation at 37°C. To over express TcTS, 0.5 mM isopropyl-β-D-thiogalactopyranoside (IPTG, Sigma) was added and growth was continued with constant agitation at 18°C, for 12–16 hs. Harvested cells were conserved at −80°C until purification. Bacterial cells were resuspend in 20 mM Tris-HCl pH 8.0, 0.5 M NaCl, 0.5% triton, 100 µg/ml DNase I, 1 mM phenylmethanesulfonyl fluoride (Sigma) and sonicated several times to reduce viscosity in a Brandson 450 sonicator. Supernatant was ultracentrifugated (45000 rpm, 45 min in a 70Ti rotor), filtered through 0.22 µm membrane filter and subjected to a Ni++ charged Hi-Trap chelating HP column (Amersham Pharmacia Biotech). Column was washed with 30 mM imidazole (Sigma) and elution was done using 100 mM imidazole in 20 mM Tris-HCl pH 8.0, 0.5 M NaCl. Elution peak was dialyzed against Tris-HCl pH 8.8, 30 mM NaCl, 1 mM EDTA and further purified by FPLC anionic exchange (MonoQ, Amersham Pharmacia Biotech). Elution was done applying a linear gradient of NaCl.

#### Immunization

Young male llamas (*Lama glama*) were injected at days 0, 21, 35 and 56 with 0.5 mg of recombinant TcTSs, using aluminum hydroxide as adjuvant. Before each boost, blood samples were collected and the sera were used to ascertain the immune response from the last immunization. At day 62 anticoagulated blood was collected and used for mononuclear cell isolation. All animal procedures were approved by the animal care committee of the IIB-INTECH in accordance with the guidelines laid out by the NIH regarding the care and use of animals for research.

#### Construction of the library

Mononuclear cells were isolated from llama 7006 heparinized blood by Ficoll-Hypaque (Pharmacia) gradient centrifugation. Total RNA was purified by TRIZOL reagent (Pharmacia) and subject to cDNA synthesis. To synthesize cDNA, 3 µg of RNA and 1.5 µg of oligo-dT_30_ in 7.5 µl were incubated for 10 min at 70°C and placed on ice. After a spin down, 0.4 mM dNTPs, RT buffer 1X, 200U M-MLV RT (Promega) and 25U RNAsin (Promega) were added, in a total volume of 25 µl and incubated 1 h at 42°C. The reaction was stopped at 70°C for 15 min.

cDNAs encoding an entire VHH domain and part of the hinge region were amplified by PCR using primers VH1Back-SfiI or VH6Back-SfiI in combination with primer Lamb7-NotI or Lamb8-NotI. Primer VHBack-SfiI anneals to VHHs N-terminal consensus sequences. Primers Lamb7-NotI and Lamb8-NotI hybridize to part of the short and long hinge region of the CH2 domain, respectively [44]. Their sequences are: VH1Back-SfiI: GCT GGA TTG TTA TTA CTC GCG GCC CAG CCG GCC ATG GCC CAG GTS MAR CTG CAG SAG TCW GG; VH6Back-SfiI: GCT GGA TTG TTA TTA TCT GCG GCC CAG CCG GCC ATG GCC GAT GTG CAG CTG CAG GCG TCT GGR GGA GG; Lamb7-NotI: G ATG GTG ATG ATG ATG TGC GGC CGC GCT GGG GTC TTC GCT GTG GTG CG; Lamb8-NotI: G ATG GTG ATG ATG ATG TGC GGC CGC TGG TTG TGG TTT TGG TGT CTT GGG. Restriction enzyme sequences are underlined. The resulting PCR fragments (∼450 bp) were purified from agarose gels (GFX PCR DNA & Gel band purification Kit, Pharmacia), digested sequentially with SfiI and NotI and repurified.

For phage-display library construction, 5 µg of SfiI-NotI-digested plasmid pHEN2 and digested fragment were ligated in a 1∶5 ratio during 16 hs at 16°C. Inserts were introduced between PelB leader signal and His-tag, in frame with pIII capsid protein gene in the vector pHEN2 [45]. The ligation reaction (200 µl) was purified with phenol:chloroform:isoamylic alcohol 25∶24∶1, extracted twice with chloroform and precipitated with 20 µg glycogen, 30 µl 2 M AcONa pH 5.2 and 440 µl EtOH for 24 hs at −80°C. The pellet was washed with 70% EtOH, dried, resuspended in 2 ml of H_2_O and concentrated with a centricon YM-3 (Amicon) to 15 µl in sterile milliQ water. Electrocompetent *E. coli* XL Blue MRF́cells were transformed and library size was calculated by plating aliquots on LB ampicillin agar. The library diversity was checked by sequencing.

#### Phage display

To prepare polyclonal phages, library stock or cells pre-infected with phages eluted during panning were grown until A_600nm_ 0.5 and were then infected with at least 10-fold excess of VCS helper phage (Promega) at 37°C in presence of glucose 1%. After one hour incubation, cells were washed by harvesting and resuspending the cells in glucose-free fresh culture media. After growing over night at 30°C, phages were recovered from culture supernatant by incubation at 4°C followed of precipitation in 4% PEG 8,000, 0.5 M NaCl. Pellet was resuspended in 1 ml sterile PBS/100 ml culture and used for panning. To enrich the library for the presence of *T. cruzi* trans-sialidase binders, panning was performed on 24 well culture plates (Hamilton). Wells were coated with 2 µg polyclonal rabbit anti-SAPA antibody for 16 hs at 4°C. After blocking with 3% skim milk in PBS (SM-PBS), 10 µg TcTSΔ1443-SAPA were added in 1% SM-PBS with agitation during 2 hs followed by a similar incubation with approximately 10^13^ VHH-phages. Washing was done 3 times with PBS between each step. VHH-phage binders were eluted by incubation with 100 mM glycine-HCl pH 2.2 during 10 min and immediately neutralized with 2 M Tris-HCl pH 8.0. This procedure was followed by amplification of the eluted phages and repeated 2 times. Phage titers of input and output at all steps were estimated by enumeration of ampicillin resistant colonies obtained from TG1 cells infected with different phage dilutions (Table 1).

To evaluate the enrichment during panning cycles, the capacity to bind TcTS of randomly chosen clones from each round was tested by phage-ELISA. Wells without immobilized protein were used as non-specific binding control (NSB). Unexpectedly, the proportion of binders decreased during successive rounds of panning due to negative selection (Table 1). Given this fact, we decided to work with phages from the first round of panning.

#### Production of individual VHH-phages

Plates with individual clones in culture medium with glucose were grown 3 hs at 37°C with agitation, and super infected with VCS helper phage for 30 min. Cells were pelleted, resuspended in the same medium without glucose and incubated for 16 hs at 30°C. The supernatants containing the VHH-phages were used for ELISA or TIA assays.

#### Elisa

Plates were sensibilized over night with 200 ng/well of polyclonal rabbit anti-SAPA antibody. After blocking with 3% SM-PBS, 250 ng TcTSΔ1443-SAPA were added in 1% SM-PBS during 2 hs. Culture supernatant containing phages expressing VHHs were diluted twofold in 2% SM-PBS and added for 2 hs. Bound phages were washed with PBS-Tween and revealed with mouse anti-M13 conjugated to HRP (Pharmacia) dilution 1/2,000. Substrate (OPD, Sigma) was added, reaction was stopped with 2 M H_2_SO_4_ and A_495nm_ was measured on an ELISA reader (Σ960 Metertech Inc.).

#### Production and purification of soluble VHHs

Phagemid DNAs recovered from six isolated clones were transformed into *E. coli* HB2151 cells. These cells are unable to suppress the amber stop codon between the cloned VHH and gene III, producing soluble VHH fragments tagged with C-terminal 6xHis upon induction with IPTG. After 4 hs induction with 1 mM IPTG, the expressed proteins were extracted from the periplasmic space through osmotic shock passing from 500 mM to 125 mM sucrose solution in buffer 200 mM Tris-HCl, 0.5 mM EDTA pH 8. The obtained VHHs have an apparent MW between 16,000 to 18,000 Da. Further purification of soluble protein was achieved on Hi-Trap chelating HP column (Pharmacia). Column was washed with 10 mM imidazole (Sigma) 0.3 M NaCl in sodium phosphate pH 8.0 and elution was done using 250 mM imidazole in the same buffer.

#### Trans-sialidase activity assay

Trans-sialidase activity was measured as the transfer of sialic acid from 1 mM sialyl-α-(2-3)-lactose (Sigma) to 12 µM [D-glucose-1-^14^C] lactose (55 mCi/mmol) (Amersham), by 0.5 ng of purified TcTS enzyme in 30 µl of 20 mM Hepes-Na (pH 7.5), 0.2% BSA, 30 mM NaCl. After 60 min at 25°C, the reaction was stopped by dilution with 1 ml of water. QAE-Sephadex (Amersham Pharmacia Biotech) was added and the resin was washed twice with water. Negative charged compounds were eluted with 0.8 ml of 1 M NaCl and quantified in a WinSpectral 1414 liquid scintillation counter (Wallac). When required, the purified enzyme was diluted in the reaction buffer before use (0.5 ng of TcTS611/2, rendered ∼4000 cpm per hour).

#### Trans-sialidase Inhibition Assay (TIA)

Culture supernatants from transformed HB2151 clones, purified phages, llama serum or purified VHHs, were assayed for their inhibitory activity on TcTS. Purified recombinant TcTS enzyme or TcTS derived from trypomastigotes (similar to the infective form of the parasite present in the mammalian host) were preincubated for 30 min with the sample to be tested, and the remnant ability to transfer the sialyl residues from sialyllactose to [d-glucose-1-^14^C] lactose was evaluated as described above. The different quantities used of each sample (llama serum, phage-VHHs or purified VHHs) are indicated in the corresponding figure. Results are expressed in percentage of inhibition of trans-sialidase activity. Reaction measured without addition of any inhibitor was considered as 0% of inhibition (100% of enzymatic activity).

#### Dot spot assay

Recombinant proteins (300 ng each) were spotted onto nitrocellulose membrane as indicated in Figure 3C. Filters were blocked for 2 hs with 5% SM-TBS, and when indicated, probed with a second purified protein for assessment of interactions. They were subsequently incubated with different antisera for 1 h (mouse anti-histidine serum (Sigma) at 1∶500 dilution or rabbit anti-TcTS serum at 1∶1,000 dilution) and washing 3 times with TBS after each treatment. Filters were processed using anti-mouse or rabbit horseradish peroxidase (HRP)-conjugated secondary antibodies (Gibco) at 1∶8,000 dilution, and positive signals were revealed by chemiluminescence (Super Signal West Pico Chemiluminescent, Pierce).

#### Affinity measurements

Kinetic analyses of the interactions were determined with IAsys Biosensor instrument (ThermoLabSystems). Each purified monoclonal VHH was immobilized in 10 mM sodium acetate pH 5.0 on a carboxymethylated dextrane layer using EDC/NHS chemistry following the manufacture instructions. 10–30 ng of VHH were immobilized. For kinetic constant determination, dilutions of TcTS in PBS-0.05% Tween were added to the cuvette. Binding traces were recorded for at least 7 different concentrations. Measurements in present of DANA were done adding DANA to the cuvette before TcTS. The addition of DANA does not induce any change in the signal. Association and dissociation rate constants were calculated using the FASTFIT software.

#### Immunoprecipitation of natural TcTS

Crude extracts of 2×10^8^ Cl-Brener trypomastigotes were incubated with purified anti-SAPA antibodies raised in mice (8 µg, ON, 4°C) followed by protein A-Sepharosa beads (75 µl, 4 hs, 4°C, Sigma). Beads were washed and conserved in Tris-HCl 20 mM, pH 7.6.

#### Deglycosylation of TcTS

PNGase F eliminates the entire N-glycosylation trees from GlcNAc of asparagine residues. Immunoprecipitated TcTS was incubated for 48 hs, at 37°C, in presence of PNGaseF (Biolabs) or in absence as a control of possible activity losses. Natural TcTS, deglycosylated or not, were diluted to rendering between 2000–3000 CPM/h (100% of activity) and assayed by TIA.

#### Cloning of Trypomastigote TcTSs

cDNA or Genomic DNA from CL-Brener trypomastigotes were amplified by PCR using primers designed to amplify the entire globular core of TcTS gene family. Their sequences are *LAPTSTE*: GGA ATT CGC TAG CCT GGC ACC CGG ATC GAG CCG A (carrying EcoRI–NheI restriction sites), *TGA:* GTG GAA TTC AGG CAC TCG TGT CGC TGC TGC TGT C (carrying EcoRI restriction site) or alternatively *3′ SAPA:* CAG CAG CAA AGC ACC CGC AC. The PCR product, containing the intrinsic variability of TcTS family, render a single band (∼2000 bp), which was cloned in pGEM-T Easy vector system (Promega) to further sequencing and selection of putative active TcTS (criteria based on presence of Tyr342) [16]. Individual chosen clones were subcloned in pTrcHisA (Invitrogen) using NheI–EcoRI restriction sites. TcTs were induced and purified as described above. The activity was assayed, linear condition of each TcTS clone concentration was confirmed before inhibition assay take place.

Data Bank accession codes- Sequences of the four TcTS strong inhibitor VHH clones have been deposited in the GenBank, with accession numbers from DQ315483 to DQ315486. Active TcTS clones from *T. cruzi* Cl-Brener strain have been deposited in the GenBank, with accession numbers from EU805797 to 805801.

## Supporting Information

Table S1 Identity index between amino acid sequences of different TcTS clones from *T. cruzi* CL-Brener strain
(0.11 MB DOC)

Figure S1 Deglycosylation of immunoprecipitated natural TcTS with PNGasa F under non-denatured conditions. (A) Coomasie blue stained SDS-PAGE. The two left lanes show the results of SBA glycoprotein (soybean agglutinin) used as a control of PNGasa F activity. In the right lanes, heavy chain (hc) corresponding to mouse anti-SAPA used to immunoprecipitate natural TcTS population from trypomastigotes, present in the same sample used in experiment indicated in panel B, that showed a lower molecular weight after treatment with PNGasa F. Due to the low amount of immunoprecipitated protein, TcTS was not detectable in coomasie blue stained gel. Panel (B) shows a Western blot of immunoprecipitated TcTS, incubated with anti-SAPA serum raised in mouse and revealed with the corresponding HRP-conjugated-anti-serum for chemiluminescence generation. The arrow is to indicate the TcTS band with a stronger signal (TcTS display several bands in Western blot) and that has an apparent lower molecular weight after PNGase F treatment. (+) indicates incubation with PNGase F and (−) indicates incubation in the absence of PNGase F.
(3.18 MB TIF)

Figure S2 Deduced amino acid sequences of entire globular core of TcTSs, without SAPA repeats, cloned from *T. cruzi* Cl-Brener strain. All sequences start with a leucine, that is the first amino acid residue in the mature natural protein [47]. Amino acidic residues differing to those present in TcTS611/2 clone are boxed. Asterisk indicates the putative N-glycosilation site near the active site, as predicted by NetNglyc 1.0 Server (www.cbs.dtu.dk). Alignment was performed by http://workbench.sdsc.edu.
(17.39 MB TIF)

## References

[1] Gupta S (2005). Parasite immune escape: new views into host-parasite
interactions.. Curr Opin Microbiol.

[2] Barrett MP, Burchmore RJ, Stich A, Lazzari JO, Frasch AC (2003). The trypanosomiases.. Lancet.

[3] Buscaglia CA, Campo VA, Frasch AC, Di Noia JM (2006). *Trypanosoma cruzi* surface mucins: host-dependent
coat diversity.. Nat Rev Microbiol.

[4] Schenkman S, Jiang MS, Hart GW, Nussenzweig V (1991). A novel cell surface trans-sialidase of *Trypanosoma
cruzi* generates a stage-specific epitope required for invasion of
mammalian cells.. Cell.

[5] Tomlinson S, Raper J (1996). The lysis of *Trypanosoma brucei brucei* by human
serum.. Nat Biotechnol.

[6] Frasch AC (2000). Functional diversity in the trans-sialidase and mucin families in
*Trypanosoma cruzi.*. Parasitol Today.

[7] Amaya MF, Buschiazzo A, Nguyen T, Alzari PM (2003). The high resolution structures of free and inhibitor-bound
*Trypanosoma rangeli* sialidase and its comparison with
T. cruzi trans-sialidase.. J Mol Biol.

[8] Buschiazzo A, Amaya MF, Cremona ML, Frasch AC, Alzari PM (2002). The crystal structure and mode of action of trans-sialidase, a
key enzyme in *Trypanosoma cruzi* pathogenesis.. Mol Cell.

[9] Buschiazzo A, Tavares GA, Campetella O, Spinelli S, Cremona ML (2000). Structural basis of sialyltransferase activity in trypanosomal
sialidases.. Embo J.

[10] Paris G, Ratier L, Amaya MF, Nguyen T, Alzari PM (2005). A sialidase mutant displaying trans-sialidase activity.. J Mol Biol.

[11] Frasch AC (1994). Trans-sialidase, SAPA amino acid repeats and the relationship
between *Trypanosoma cruzi* and the mammalian host.. Parasitology.

[12] Buscaglia CA, Alfonso J, Campetella O, Frasch AC (1999). Tandem amino acid repeats from *Trypanosoma cruzi*
shed antigens increase the half-life of proteins in blood.. Blood.

[13] Buscaglia CA, Campetella O, Leguizamon MS, Frasch AC (1998). The repetitive domain of *Trypanosoma cruzi*
trans-sialidase enhances the immune response against the catalytic domain.. J Infect Dis.

[14] Affranchino JL, Ibanez CF, Luquetti AO, Rassi A, Reyes MB (1989). Identification of a *Trypanosoma* cruzi antigen
that is shed during the acute phase of Chagas' disease.. Mol Biochem Parasitol.

[15] Leguizamon MS, Campetella O, Russomando G, Almiron M, Guillen I (1994). Antibodies inhibiting *Trypanosoma cruzi*
trans-sialidase activity in sera from human infections.. J Infect Dis.

[16] Cremona ML, Campetella O, Sanchez DO, Frasch AC (1999). Enzymically inactive members of the trans-sialidase family from
*Trypanosoma cruzi* display beta-galactose binding
activity.. Glycobiology.

[17] Schenkman S, Eichinger D, Pereira ME, Nussenzweig V (1994). Structural and functional properties of
*Trypanosoma* trans-sialidase.. Annu Rev Microbiol.

[18] Mucci J, Hidalgo A, Mocetti E, Argibay PF, Leguizamon MS (2002). Thymocyte depletion in *Trypanosoma cruzi*
infection is mediated by trans-sialidase-induced apoptosis on nurse cells
complex.. Proc Natl Acad Sci U S A.

[19] Todeschini AR, Mendonca-Previato L, Previato JO, Varki A, van Halbeek H (2000). Trans-sialidase from *Trypanosoma cruzi* catalyzes
sialoside hydrolysis with retention of configuration.. Glycobiology.

[20] Agusti R, Paris G, Ratier L, Frasch AC, de Lederkremer RM (2004). Lactose derivatives are inhibitors of *Trypanosoma
cruzi* trans-sialidase activity toward conventional substrates in
vitro and in vivo.. Glycobiology.

[21] Mucci J, Risso MG, Leguizamon MS, Frasch AC, Campetella O (2006). The trans-sialidase from *Trypanosoma cruzi*
triggers apoptosis by target cell sialylation.. Cell Microbiol.

[22] Leguizamon MS, Campetella OE, Gonzalez Cappa SM, Frasch AC (1994). Mice infected with *Trypanosoma cruzi* produce
antibodies against the enzymatic domain of trans-sialidase that inhibit its
activity.. Infect Immun.

[23] Pereira-Chioccola VL, Schenkman S, Kloetzel JK (1994). Sera from chronic Chagasic patients and rodents infected with
*Trypanosoma cruzi* inhibit trans-sialidase by
recognizing its amino-terminal and catalytic domain.. Infect Immun.

[24] Leguizamon MS, Russomando G, Luquetti A, Rassi A, Almiron M (1997). Long-lasting antibodies detected by a trans-sialidase inhibition
assay of sera from parasite-free, serologically cured chagasic patients.. J Infect Dis.

[25] Tribulatti MV, Mucci J, Van Rooijen N, Leguizamon MS, Campetella O (2005). The trans-sialidase from *Trypanosoma cruzi*
induces thrombocytopenia during acute Chagas' disease by reducing
the platelet sialic acid contents.. Infect Immun.

[26] Lauwereys M, Arbabi Ghahroudi M, Desmyter A, Kinne J, Holzer W (1998). Potent enzyme inhibitors derived from dromedary heavy-chain
antibodies.. Embo J.

[27] Hamers-Casterman C, Atarhouch T, Muyldermans S, Robinson G, Hamers C (1993). Naturally occurring antibodies devoid of light chains.. Nature.

[28] Transue TR, De Genst E, Ghahroudi MA, Wyns L, Muyldermans S (1998). Camel single-domain antibody inhibits enzyme by mimicking
carbohydrate substrate.. Proteins.

[29] Pitcovsky TA, Mucci J, Alvarez P, Leguizamon MS, Burrone O (2001). Epitope mapping of trans-sialidase from *Trypanosoma
cruzi* reveals the presence of several cross-reactive determinants.. Infect Immun.

[30] Pitcovsky TA, Buscaglia CA, Mucci J, Campetella O (2002). A functional network of intramolecular cross-reacting epitopes
delays the elicitation of neutralizing antibodies to *Trypanosoma
cruzi* trans-sialidase.. J Infect Dis.

[31] Buschiazzo A, Campetella O, Frasch AC (1997). *Trypanosoma rangeli* sialidase: cloning,
expression and similarity to T. cruzi trans-sialidase.. Glycobiology.

[32] Paris G, Cremona ML, Amaya MF, Buschiazzo A, Giambiagi S (2001). Probing molecular function of trypanosomal sialidases: single
point mutations can change substrate specificity and increase hydrolytic
activity.. Glycobiology.

[33] Amaya MF, Watts AG, Damager I, Wehenkel A, Nguyen T (2004). Structural insights into the catalytic mechanism of
*Trypanosoma cruzi* trans-sialidase.. Structure.

[34] Rastam L, Lindberg G, Folsom AR, Burke GL, Nilsson-Ehle P (1996). Association between serum sialic acid concentration and carotid
atherosclerosis measured by B-mode ultrasound. The ARIC Investigators.
Atherosclerosis Risk in Communities Study.. Int J Epidemiol.

[35] Craig PO, Berguer PM, Ainciart N, Zylberman V, Thomas MG (2005). Multiple display of a protein domain on a bacterial polymeric
scaffold.. Proteins.

[36] Harmsen MM, van Solt CB, Hoogendoorn A, van Zijderveld FG, Niewold TA (2005). Escherichia coli F4 fimbriae specific llama single-domain
antibody fragments effectively inhibit bacterial adhesion in vitro but
poorly protect against diarrhoea.. Vet Microbiol.

[37] Conrath KE, Lauwereys M, Galleni M, Matagne A, Frere JM (2001). Beta-lactamase inhibitors derived from single-domain antibody
fragments elicited in the camelidae.. Antimicrob Agents Chemother.

[38] Desmyter A, Spinelli S, Payan F, Lauwereys M, Wyns L (2002). Three camelid VHH domains in complex with porcine pancreatic
alpha-amylase. Inhibition and versatility of binding topology.. J Biol Chem.

[39] Atwood JA, Weatherly DB, Minning TA, Bundy B, Cavola C (2005). The *Trypanosoma cruzi* proteome.. Science.

[40] Fontanella GH, De Vusser K, Laroy W, Daurelio L, Nocito AL (2008). Immunization with an engineered mutant trans-sialidase highly
protects mice from experimental *Trypanosoma cruzi*
infection: a vaccine candidate.. Vaccine.

[41] Hoft DF, Eickhoff CS, Giddings OK, Vasconcelos JR, Rodrigues MM (2007). Trans-sialidase recombinant protein mixed with CpG
motif-containing oligodeoxynucleotide induces protective mucosal and
systemic *Trypanosoma cruzi* immunity involving CD8+
CTL and B cell-mediated cross-priming.. J Immunol.

[42] Tarleton RL (2007). Immune system recognition of *Trypanosoma cruzi*.. Curr Opin Immunol.

[43] Vasconcelos JR, Hiyane MI, Marinho CR, Claser C, Machado AM (2004). Protective immunity against *Trypanosoma cruzi*
infection in a highly susceptible mouse strain after vaccination with genes
encoding the amastigote surface protein-2 and trans-sialidase.. Hum Gene Ther.

[44] Muyldermans S, Atarhouch T, Saldanha J, Barbosa JA, Hamers R (1994). Sequence and structure of VH domain from naturally occurring
camel heavy chain immunoglobulins lacking light chains.. Protein Eng.

[45] Griffiths AD, Williams SC, Hartley O, Tomlinson IM, Waterhouse P (1994). Isolation of high affinity human antibodies directly from large
synthetic repertoires.. Embo J.

[46] Lefranc MP, Giudicelli V, Ginestoux C, Bodmer J, Muller W (1999). IMGT, the international ImMunoGeneTics database.. Nucleic Acids Res.

[47] Pollevick GD, Sanchez DO, Campetella O, Trombetta S, Sousa M (1993). Members of the SAPA/trans-sialidase protein family have identical
N-terminal sequences and a putative signal peptide.. Mol Biochem Parasitol.

