# Supplementary material for: Relevance of the Diversity among Members of the Trypanosoma Cruzi Trans-Sialidase Family Analyzed with Camelids Single-Domain Antibodies
Source: PLoS One. 2008 Oct 24;3(10):e3524. doi: 10.1371/journal.pone.0003524 (PMC2568053; doi:10.1371/journal.pone.0003524)
Supplement: Table S1 — Identity index between amino acid sequences of different TcTS clones from T. cruzi CL-Brener strain (0.11 MB DOC) [file pone.0003524.s001.doc]

TABLE S1. *Identity index between amino acid sequences of different TcTS clones from T. cruzi CL-Brener strain*

| Protein | TcTS-46 | TcTS-49 | TcTS-24 | TcTS-15 | TcTS-12 | TcTS-611/2 |
| --- | --- | --- | --- | --- | --- | --- |
| TcTS-46 | 100.0 | 98.8 | 88.9 | 89.1 | 89.7 | 90.6 |
| TcTS-49 |  | 100.0 | 89.4 | 89.5 | 90.2 | 90.3 |
| TcTS-24 |  |  | 100.0 | 99.5 | 98.8 | 95.9 |
| TcTS-15 |  |  |  | 100.0 | 98.9 | 96.1 |
| TcTS-12 |  |  |  |  | 100.0 | 96.7 |
| TcTS-611/2 |  |  |  |  |  | 100.0 |
