# Supplementary figures and images for: Relevance of the Diversity among Members of the Trypanosoma Cruzi Trans-Sialidase Family Analyzed with Camelids Single-Domain Antibodies
Source: PLoS One. 2008 Oct 24;3(10):e3524. doi: 10.1371/journal.pone.0003524 (PMC2568053; doi:10.1371/journal.pone.0003524)

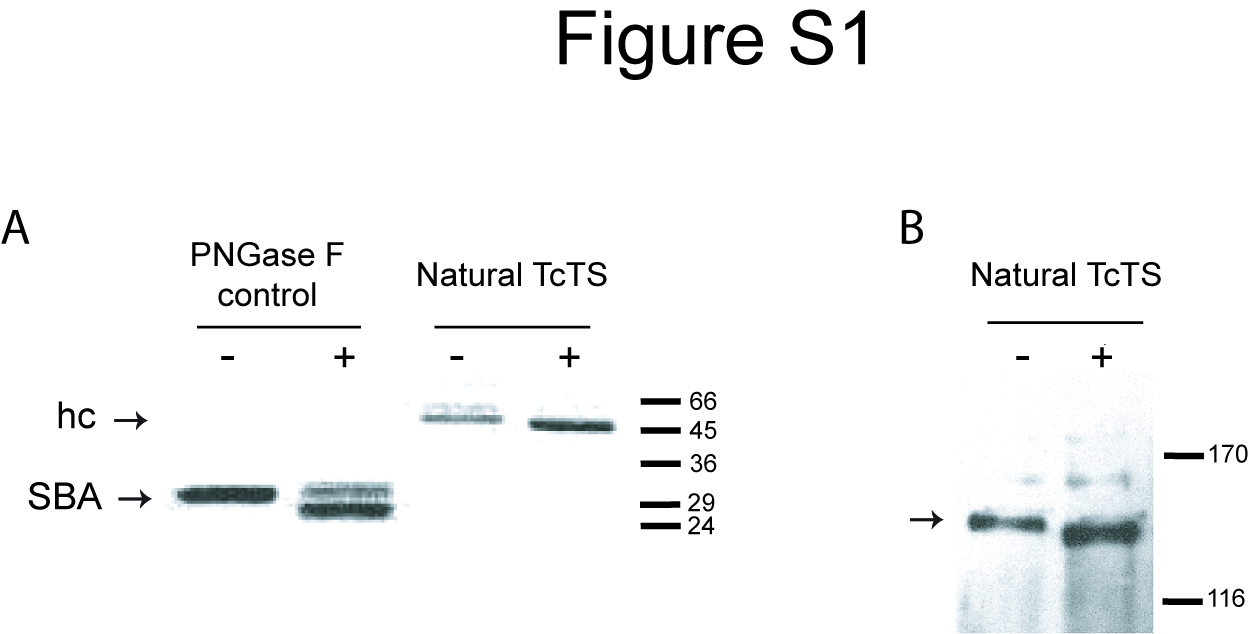

Supplement: Figure S1 — Deglycosylation of immunoprecipitated natural TcTS with PNGasa F under non-denatured conditions. (A) Coomasie blue stained SDS-PAGE. The two left lanes show the results of SBA glycoprotein (soybean agglutinin) used as a control of PNGasa F activity. In the right lanes, heavy chain (hc) corresponding to mouse anti-SAPA used to immunoprecipitate natural TcTS population from trypomastigotes, present in the same sample used in experiment indicated in panel B, that showed a lower molecular weight after treatment with PNGasa F. Due to the low amount of immunoprecipitated protein, TcTS was not detectable in coomasie blue stained gel. Panel (B) shows a Western blot of immunoprecipitated TcTS, incubated with anti-SAPA serum raised in mouse and revealed with the corresponding HRP-conjugated-anti-serum for chemiluminescence generation. The arrow is to indicate the TcTS band with a stronger signal (TcTS display several bands in Western blot) and that has an apparent lower molecular weight after PNGase F treatment. (+) indicates incubation with PNGase F and (−) indicates incubation in the absence of PNGase F. (3.18 MB TIF) [file pone.0003524.s002.tif]

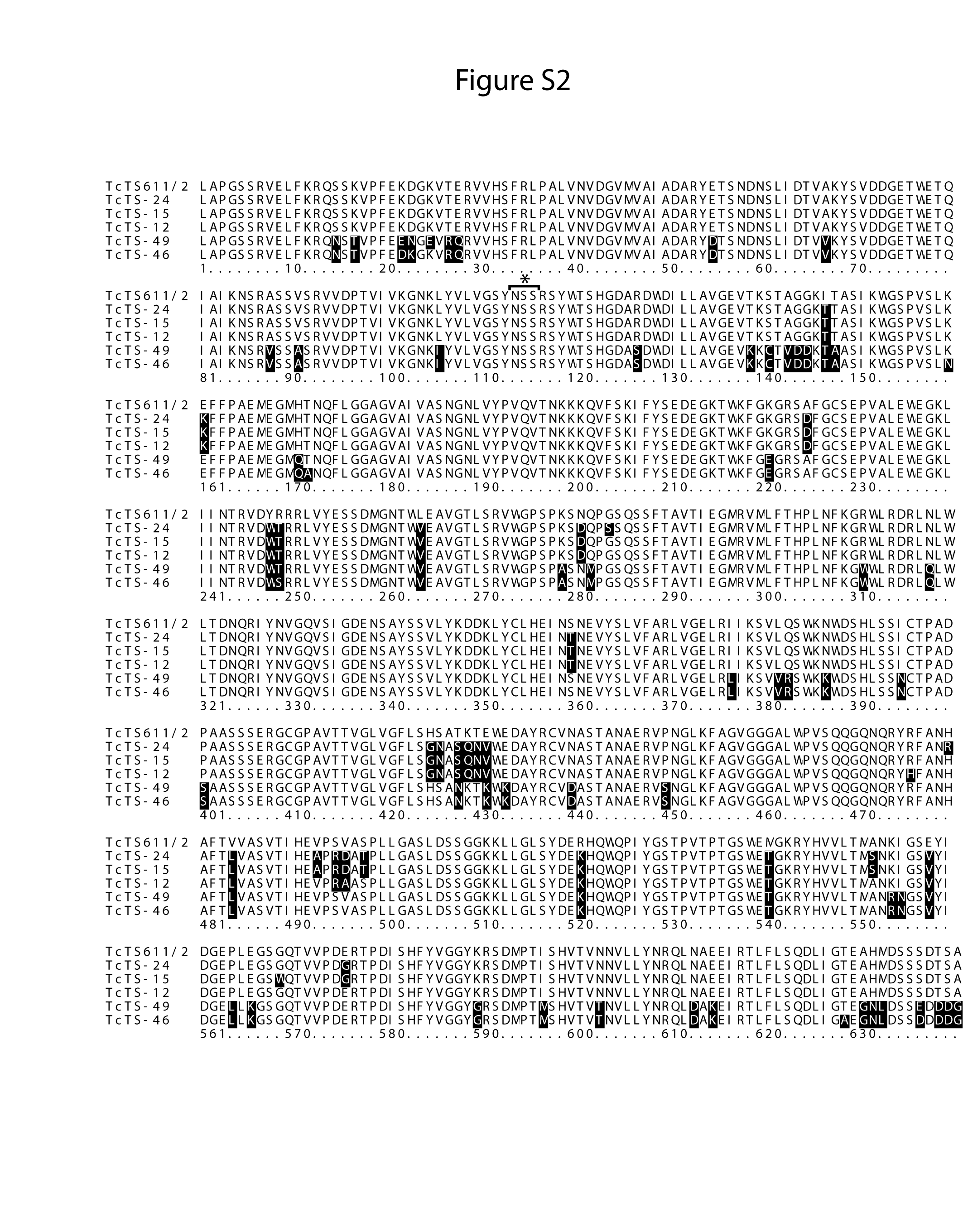

Supplement: Figure S2 — Deduced amino acid sequences of entire globular core of TcTSs, without SAPA repeats, cloned from T. cruzi Cl-Brener strain. All sequences start with a leucine, that is the first amino acid residue in the mature natural protein [47]. Amino acidic residues differing to those present in TcTS611/2 clone are boxed. Asterisk indicates the putative N-glycosilation site near the active site, as predicted by NetNglyc 1.0 Server (www.cbs.dtu.dk). Alignment was performed by http://workbench.sdsc.edu. (17.39 MB TIF) [file pone.0003524.s003.tif]
